# Role of Arterial Hypertension and Hypertension-Mediated Organ Damage in Cardiotoxicity of Anticancer Therapies

**DOI:** 10.1007/s11897-023-00590-5

**Published:** 2023-01-25

**Authors:** Giacomo Tini, Giuliano Tocci, Allegra Battistoni, Matteo Sarocchi, Camilla Pietrantoni, Domitilla Russo, Beatrice Musumeci, Carmine Savoia, Massimo Volpe, Paolo Spallarossa

**Affiliations:** 1 Division of Cardiology, Department of Clinical and Molecular Medicine, University of Rome Sapienza, Sant’Andrea Hospital, Rome, Italy; 2 IRCCS San Raffaele Pisana, Rome, Italy; 3 Cardiovascular Disease Unit, IRCCS Ospedale Policlinico San Martino–IRCCS Italian Cardiology Network, Genoa, Italy

**Keywords:** Arterial hypertension, Arterial hypertension-mediated organ damage, Anthracycline, Anti-VEGF, Cardiotoxicity, Cardio-oncology

## Abstract

**Purpose of the Review:**

Arterial hypertension (AH) is the most common cardiovascular (CV) risk factor in the community and in oncologic patients. It also represents the most important CV condition predisposing to anticancer treatment-related cardiotoxicity. This risk is heightened in the presence of cardiac AH-mediated organ damage (HMOD). Influence of AH and HMOD on the development of cardiotoxicity will be reviewed, with a focus on specific scenarios and implications for management of oncologic patients.

**Recent Findings:**

Not adequately controlled AH before or during anticancer treatments and/or development of AH during or after completion of such therapies have detrimental effects on the clinical course of oncologic patients, particularly if HMOD is present.

**Summary:**

As overlooking CV health can jeopardize the success of anticancer treatments, the goal for clinicians caring for the oncologic patient should include the treatment of AH and HMOD.

## Introduction

Cardiovascular (CV) adverse events related to anticancer therapies are defined as *cardiotoxicity*. This term represents a heterogeneous group of conditions including but not limited to left ventricular dysfunction (LVD) and overt heart failure (HF), myocarditis, venous thromboembolism, arterial occlusive events, arrhythmias, and arterial hypertension (AH) [1, 2•, 3•, 4, 5]. Occurrence of cardiotoxicity mainly depends on two factors: the type of anticancer treatment with its inherent toxicity and the individual CV risk profile [6•, 7, 8].

Beside cardiotoxicity, cancer patients are also at increased risk of developing CV disease (CVD) in the long term after completion of anticancer treatments [9], and such risk is heightened in the presence of a worse CV risk profile [10, 11••]. The burden and need for treatment of CV comorbidities in cancer patients, once overlooked, have thus been recognized as essential for an integrated strategy of CV prevention in the field of cardio-oncology [1, 12, 13].

## The Importance of Arterial Hypertension in Cancer Patients

AH is the most common comorbidity in cancer patients, found in about 35–38% of the general oncologic population [14–16]. It is typically considered the most important CV factor favoring cardiotoxicity, in particular LVD [17]. AH is known to have per se a detrimental CV effect and often clusters with other CV risk factors, thus worsening the overall individual risk profile [18]. This is true also in oncologic patients. For example, in an administrative database study on oncologic patients eligible for anti-vascular endothelial growth factor (anti-VEGF) therapies, those with AH had also more commonly other CV risk factors and comorbidities [19]. Moreover, patients affected by AH prior to starting anticancer therapy are at higher odds of developing an elevation of blood pressure (BP) values as cardiotoxicity [17].

For these reasons, an aggressive and careful treatment of AH in oncologic patients has been advocated, yet it is still often overlooked [17, 20••, 21]. Furthermore, in the oncologic setting, CV risk factors are usually defined based on the clinical history (i.e., present vs. absent) regardless of whether they are controlled or not, thus hindering the possibility of assessing their true influence on the risk of cardiotoxicity [12, 22••]. Therefore, a close collaboration between oncologists and cardiologists is highly recommended before initiation of anticancer treatments.

It is important to point out that such considerations refer to the whole spectrum of anticancer treatments and not only to classic chemotherapy. Contemporary anticancer treatments include, for example, hormone therapy for a variety of cancers. AH plays an important role also in these settings. Indeed, hormone treatments may cause elevation of BP values, as in the case of abiraterone for prostate cancer [23], or worsen the overall CV profile, above which AH may be a superimposed stressor, as in the case of hormone therapy for breast cancer [24, 25].

The adverse pathological effects of AH are enhanced in the presence of the so-called AH-mediated organ damage (HMOD). The development of HMOD in the vessels, heart [26, 27••, 28], and kidney [29, 30] is related to adverse outcomes in the general AH population and contributes further to worsen the overall CV profile [31] both in men and women [32]. Moreover, despite the prevalence of HMOD associated with increasing BP values, it can be found across the whole “spectrum” of AH (i.e., not only in long-standing AH or severely uncontrolled AH) and in each BP category its presence increases CV risk significantly [33••]. Thus, also in oncologic individuals, the presence of HMOD needs to be checked, as it represents a proxy of even greater risk for cardiotoxicities [34].

The influence of AH and HMOD on the development of cardiotoxicity is not limited to the time when anticancer therapies are delivered. In cancer survivors who had received cardiotoxic drugs such as anthracyclines, cardiotoxicity may occur even years after end of treatment, and its development may be triggered by various stressors including AH [2•].

Therefore, in oncologic patients, both pre-existing and post hoc AH (considering “index time” the administration of anticancer therapies) exert severe and detrimental effects.

## When Arterial Hypertension Represents Cardiotoxicity: the Case of Anti-VEGF Agents

Anti-VEGF agents comprise three groups of drugs: humanized monoclonal antibodies that directly bind to VEGF, tyrosine kinase inhibitors (TKIs), and soluble decoy receptors acting as “VEGF traps” [35]. Virtually, all patients treated with anti-VEGF agents develop an increase in BP values, and adverse events related to AH may occur in up to 60% of cases depending on the specific agent (Table 1) [17, 36]. AH is mainly an “on-target” effect of anti-VEGF drugs, meaning that the rise in BP values is due to the same mechanisms by which these agents exert their anticancer effect. In particular, by inhibiting VEGF receptor 2, these anticancer agents determine a reduction in nitric oxide (NO) production in vessels, which in turn causes vasoconstriction, augmentation of peripheral resistances, and overproduction of reactive oxygen species [37, 38]. Moreover, inhibition of VEGF also induces kidney glomerular lesion, proteinuria and worsening renal function, and even a direct myocardial damage [17, 39, 40]. These latter events are instead due to “off-target” effects. Anti-VEGF agents also increase levels of endothelin 1, a molecule with vasoconstrictive effect, which elicits endothelial cell apoptosis, resulting in microcapillary rarefactions, and induces renal thrombotic microangiopathy [17].

**Table 1:** Anti-VEGF agents and related incidence of arterial hypertension

Anti-VEGF agent	Therapeutic target	Incidence of arterial hypertension
Bevacizumab	VEGF ligand	22–24%
Sunitinib	VEGFR, PDGFR, KIT, FLT3, CSR, RET	15–34%
Sorafenib	VEGFR, PDGFR, KIT, FLT3, RET	17–29%
Axitinib	VEGFR	40%
Pazopanib	VEGFR, PDGFR, FGFR, KIT, Itk, Lck, c-FMS	36–46%
Ponatinib	VEGFR, PDGFR, FGFR, EPH, BCR-ABL, KIT, FLT3, RET, Src, TIE2	67%
Regorafenib	VEGFR, PDGFR, FGFR, KIT, RET, BRAF	28–48%
Cabozantinib	VEGFR, KIT, FLT3, RET, MET, TRKB, AXL, TIE2	32–37%
Vandetanib	VEGFR, EGFR, RET	24%

At the clinical level, thus, anti-VEGF agents are well-known to cause AH and AH-related disorders. Trials and real-world data have indeed shown that these anticancer drugs are associated with renal adverse events and HF [17, 35, 41]. AH due to anti-VEGF, and consequent HMOD, have a significant clinical impact, as these CV events may be severe and cause discontinuation of the anticancer treatment [42]. Since most anti-VEGF therapies are delivered in advanced cancer settings, interruption of treatment may have important prognostic implications.

The most important risk factor for BP increase due to anti-VEGF agents is preexisting AH [43]. Accordingly, the risk of AH-related adverse events due to anti-VEGF is heightened in the presence of preexisting AH and HMOD, both renal and cardiac [1, 35, 44]. Thus, caution is required if a patient scheduled to receive anti-VEGF agents has a history of chronic kidney disease, proteinuria, myocardial infarction or HF. The quick and uncontrolled increase in BP that frequently occurs with these drugs may rapidly decompensate the preexisting clinical status [17].

Nevertheless, AH due to anti-VEGF agents appears easily manageable [17, 45]. Despite some degree of damage due to the intrinsic toxicity of these drugs being hardly avoidable, it has been shown that if the increase in BP is well controlled, the added value of preexisting HMOD onto the risk of renal and CV adverse events may be attenuated [22••, 46]. We have previously shown that a baseline cardio-oncologic thorough CV assessment of cancer patients scheduled to receive anti-VEGF agents was instrumental to optimize their CV profile (given the high prevalence of risk factors, frequently not adequately controlled) and to set up AH management. This approach consists of advising the patient and the referring oncologist regarding the possibility of BP increase and of the importance of BP control [17, 22••]. If the patient has preexisting and uncontrolled AH, therapy is optimized. In case of newly diagnosed AH, an anti-hypertensive therapy is suggested (usually with low-dose combination of angiotensin-converting enzyme inhibitors and calcium channel blockers). Consequently, we found that preexisting AH, even if not adequately controlled at baseline, and chronic kidney disease were no longer associated with the occurrence of CV and renal events during anti-VEGF treatment [22••]. Furthermore, it has been shown that ponatinib causes both AH and direct vascular damage, and patients with prior history of AH or of HMOD (especially peripheral arterial occlusive disease) have an up to twofold increased risk of CV adverse events, in particular arterial occlusive events [47–49]. Nevertheless, it has been shown that if patients with AH scheduled to receive ponatinib are strictly controlled and their BP is well treated, the risk of CV adverse events is reduced. Moreover, patients burdened by HMOD may be eligible for a ponatinib dose reduction, with maintained efficacy and higher safety [50–52].

This evidence highlights the importance of a baseline evaluation of cancer patients scheduled to receive potentially cardiotoxic treatments in order to assess and, if necessary, mitigate the individual CV risk profile [17, 53••].

## When Arterial Hypertension Triggers Cardiotoxicity: the Case of Anthracycline

**Fig. 1:**
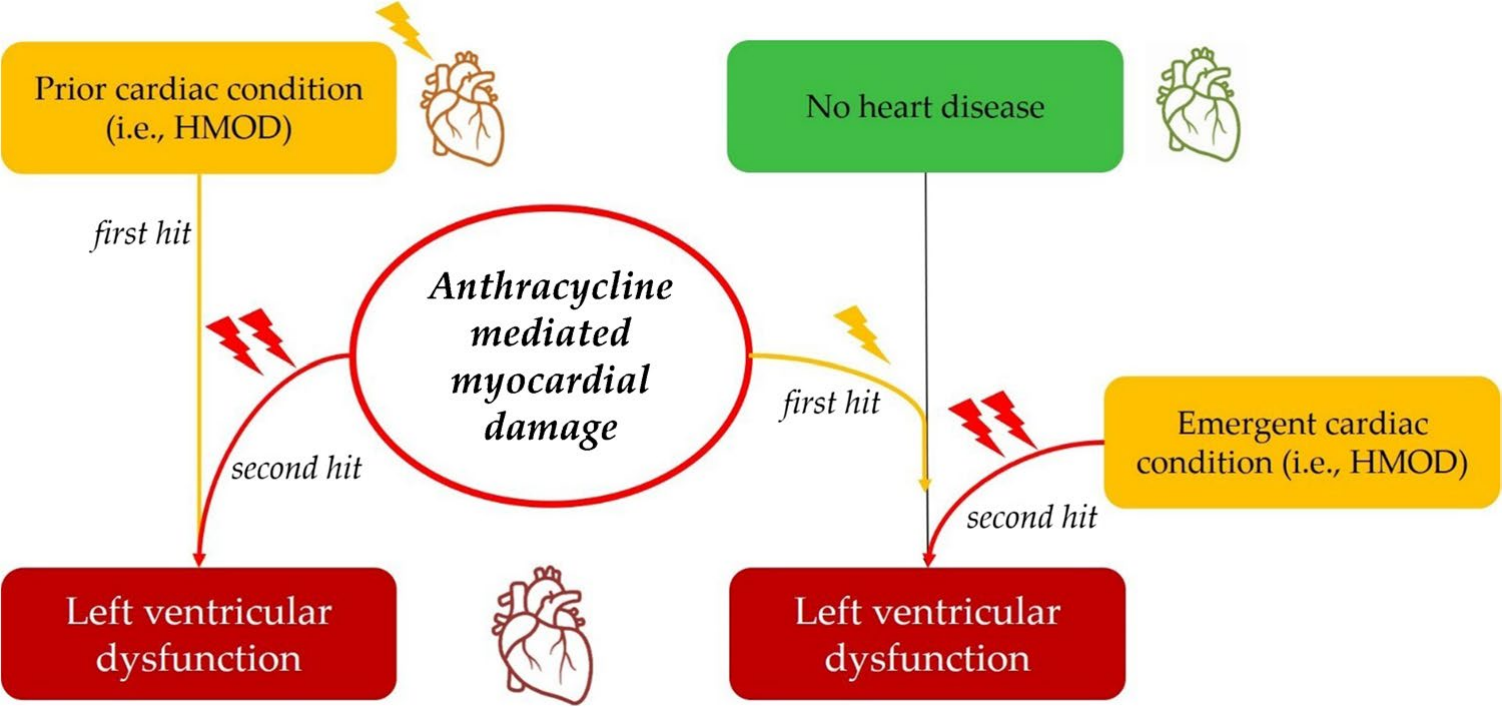
Cardiotoxicity due to anthracycline: the multiple-hit hypothesis

Cardiotoxicity due to anthracyclines occurs mainly due to three mechanisms. Traditionally, it has been related to an iron-mediated overproduction of reactive oxygen species [54]. Moreover, anthracyclines target the DNA topoisomerase II isoenzymes α and β. The latter is responsible for cardiotoxicity, since its inhibition in cardiomyocytes causes double-stranded breaks in DNA, transcriptome changes, reactive oxygen species formation, and apoptosis [55, 56]. Finally, metabolites of anthracyclines accumulate within cardiomyocytes and contribute to persisting cardiotoxic damage [57]. According to the “multiple-hit” hypothesis, cardiotoxicity due to anthracyclines occur when the direct damage of the drug, combined with other stressors (aging and comorbidities), reaches a “point-of-no-return” threshold [2•, 58]. At the clinical level, this has two main implications. First, anthracycline cardiotoxicity is amplified by CV risk factors and amplifies CV risk factor-induced cardiac damage [59] (Fig. 1). Secondly, anthracycline cardiotoxicity may occur even years after end of treatment (i.e., long-term cardiotoxicity) [2•].

Monitoring and management of CV risk profile are of primary importance in anthracycline recipients [12]. AH is recognized as the most important CV risk factor associated with anthracycline cardiotoxicity [17, 60]. Moreover, cancer patients treated with anthracycline with known AH have been reported to be more likely to undergo therapy discontinuation or delay or a reduction in anthracycline doses, with significant prognostic implications [61].

AH may trigger anthracycline cardiotoxicity both if it is pre-existing and when it develops after anticancer treatment (Fig. 1). In the first case, AH is the substrate on which anthracyclines exert their direct damage; in the second scenario, AH is the “second hit”, exacerbating the prior anthracycline effect [17]. However, while preexisting LVD and previous myocardial infarction are conditions easy to “spot,” AH may cause subtle damage to the heart. Cardiac HMOD may manifest as left ventricular hypertrophy (LVH) or as HF with preserved ejection fraction, which may be difficult to identify in inter-critical, well-compensated phases [62, 63].

In patients scheduled to receive anthracyclines, AH should be recognized as an important risk factor for cardiotoxicity [17], with such risk being further increased in the presence of HMOD. In a recent study, it has been shown that patients with AH affected by lymphoma and receiving anthracycline had a greater risk of cardiotoxicity if presenting LVH [64]. However, beside an adequate and meticulous treatment of AH, few strategies have proven beneficial for the prevention of anthracycline-induced cardiotoxicity [2•, 65]. This is a very important concept when one considers implications for long-term follow-up of cancer patients who received anthracycline therapy, and the possibility of incident new-onset AH (and even HMOD). Indeed, cancer patients with both pre-existing and post hoc CV conditions (compared to those without) have worse short-term [66] and long-term outcomes [10] after anticancer treatment completion. Similarly, CV risk factors, AH in particular, play an important role in CV event occurrence in childhood cancer survivors [67].

Thus, anthracycline recipients should be advised to continue life-long CV monitoring [1, 17]. Once cardiotoxicity has developed, cardioactive drugs as beta-blockers, angiotensin-converting enzyme inhibitors, or angiotensin receptor blockers surely may play a role in attenuating the detrimental effects of anthracyclines; however, primary prevention still represents the best way to avoid cardiotoxicity. Consistently, AH must be treated promptly, as cardiac HMOD is irreversible. In this context, cardio-oncology practice may serve as an important tool promoting CV health and prevention in the oncologic setting [7, 22••, 68].

## Practical Implications

In 2020, the Heart Failure Association of the European Society of Cardiology, together with the International Cardio-Oncology Society, published a proposal for routine assessment of CV risk in oncologic patients scheduled to receive anticancer treatments associated with cardiotoxicities [53••]. This was a welcome acknowledgement of the fact that a baseline cardio-oncology visit may provide a unique opportunity to comprehensively evaluate CV health before initiation of cancer treatment [7, 22••] for patients in whom it would otherwise be overlooked or considered too late (i.e., when cardiotoxicity has already occurred). The proposal by the Heart Failure Association and the International Cardio-Oncology Society provides charts to estimate the risk of cardiotoxicity for the main classes of anticancer therapies [53••]. The importance of CV prevention strategies, targeted at adequate control of classic CV risk factors in oncologic patients, has then furthermore stressed in the recent European Society of Cardiology guidelines on cardio-oncology published in 2022 [69••]. The guidelines recommend an aggressive treatment of CV risk factors, both during and after anticancer treatment completion, with a particular mention for AH. Indeed, guidelines remark the importance of adequate BP control, especially in oncologic patients with known AH and in those scheduled to receive anti-VEGF agents.

Yet, how to perform a baseline cardio-oncology evaluation varies taking into account several factors, including the patient status, the specific scheduled anticancer treatment, and the organization of each cardio-oncology center [17, 70]. While in the majority of cases a well-performed medical history collection, a cardiologic visit, and an ECG are largely enough for a baseline cardio-oncology evaluation, some specific cases are worthy of further attention. First, it should be kept in mind a paramount concept that holds true for each CV risk factor, and here it is reported for AH: not all patients have known AH or, if known, adequately controlled AH. The importance of a baseline evaluation stands in the fact that not only the presence versus absence of a CV risk factor is checked, but the adequate versus inadequate control of such risk factor is performed [12, 17, 22••], which is somehow more important than only knowing if a CV risk factor is present. Since HMOD may be concealed, all patients with AH (not only those symptomatic or with a prior history of CV events) should be advised to perform a comprehensive HMOD screening [17, 18, 52] if not scheduled as a routine procedure. Echocardiography may be performed in the same occasion of the visit, with a significant added value to the baseline consultation. Even though this approach may be perceived as time consuming or not cost-effective, it is reasonable to assume that a one-time-only thorough CV check-up in the oncologic setting holds great potential and may represent an investment to avoid unplanned cardiologic evaluations during anticancer treatment, with the risk of holding a therapy [7, 68]. Hence, patients with AH scheduled to receive specific anticancer treatments such as ponatinib [53••] should be checked for HMOD and, in particular, peripheral arterial occlusive disease. This would not only significantly reduce the risk of arterial occlusive events (the most frequent CV toxicity with ponatinib) but also allow to modulate the dose of the anticancer treatment based on the CV risk profile of each patient [50–52].

Thus, the baseline cardio-oncology evaluation helps to customize management of CV profile for each patient and concurrently to lower the risk for cardiotoxicities [17]. In the case of AH, its presence and, most importantly, control should be assessed; when AH is present, HMOD must be checked.

## Conclusions

AH is the most important CV condition predisposing to anticancer treatment-related cardiotoxicity. This risk is heightened in the presence of cardiac HMOD. Moreover, AH may itself be an adverse effect of anticancer treatment, leading to therapy discontinuation and poor outcomes. Therefore, the good assessment and control of CV risk profile, including the optimization of AH therapy, are of primary importance in the management of cancer patients. As overlooking CV health can jeopardize the success of anticancer treatments, the goal for clinicians caring for the oncologic patient should include the treatment of AH and HMOD.
